# Subliminal cues bias perception of facial affect in patients with social phobia: evidence for enhanced unconscious threat processing

**DOI:** 10.3389/fnhum.2014.00580

**Published:** 2014-08-04

**Authors:** Aiste Jusyte, Michael Schönenberg

**Affiliations:** ^1^Department of Clinical Psychology and Psychotherapy, University of TübingenTübingen, Germany; ^2^LEAD Graduate School, University of TübingenTübingen, Germany

**Keywords:** social anxiety, threat bias, subliminal, face perception, preattentive processing

## Abstract

Socially anxious individuals have been shown to exhibit altered processing of facial affect, especially expressions signaling threat. Enhanced unaware processing has been suggested an important mechanism which may give rise to anxious conscious cognition and behavior. This study investigated whether individuals with social anxiety disorder (SAD) are perceptually more vulnerable to the biasing effects of subliminal threat cues compared to healthy controls. In a perceptual judgment task, 23 SAD and 23 matched control participants were asked to rate the affective valence of parametrically manipulated affective expressions ranging from neutral to angry. Each trial was preceded by subliminal presentation of an angry/neutral cue. The SAD group tended to rate target faces as “angry” when the preceding subliminal stimulus was angry vs. neutral, while healthy participants were not biased by the subliminal stimulus presentation. The perceptual bias in SAD was also associated with higher reaction time latencies in the subliminal angry cue condition. The results provide further support for enhanced unconscious threat processing in SAD individuals. The implications for etiology, maintenance, and treatment of SAD are discussed.

## INTRODUCTION

According to evolutionary accounts of threat processing, affective facial expressions, especially those depicting a source of direct (anger) or indirect (fear, disgust) threat represent a class of signals relevant for survival. A great amount of empirical evidence suggests that very quick processing of threatening signals is a part of an innate functional repertoire of a healthy human brain (Williams et al., 2004; Koster et al., 2005; Bar-Haim et al., 2007; Ohrmann et al., 2007; Bishop, 2008; Bannerman et al., 2009; LoBue, 2009; Pichon et al., 2012). These results can be interpreted in terms of the preparedness theory, according to which the existence of these neuronal mechanisms are beneficial for the survival of the organism (LeDoux, 2000, 2003; Öhman et al., 2000, 2007; Öhman and Mineka, 2001a). Quick information processing may translate to a crucial temporal advantage of milliseconds to prepare for and execute a behavioral response when faced with sudden danger. Due to direct neural projections to the visual cortex, the amygdala is considered to be the key structure modulating this early processing advantage for threatening information (Morris et al., 1998, 2001; Anderson and Phelps, 2001; Vuilleumier et al., 2004). In accordance with these neuroimaging findings, prior studies have provided behavioral evidence by demonstrating that fearful cues actually enhance perceptual sensitivity (Phelps et al., 2006; Stolarova et al., 2006; Lim and Pessoa, 2008). However, the threatening character of certain stimuli does not necessarily have to be inherent, but may also acquire their aversive quality through learning experiences (Stolarova et al., 2006; Keil et al., 2007a,b). These different types of threatening stimuli may reflect qualitatively different aspects of a threat, e.g., inherent vs. acquired, which may have affect processing in different ways. Prior investigations from our work group showed that participants acquired a perceptual bias to subliminal threat only when inherently aversive stimuli (angry faces) were paired with aversive outcomes via a prior conditioning procedure (Jusyte and Schönenberg, 2013). While this initial evidence suggests that learning experiences may have enhance unconscious visual processing of threatening stimuli, it remains unclear how durable these effects may be and whether similar mechanisms can be assumed in relevant psychopathologies, such as anxiety disorders.

Generalized social anxiety disorder (SAD) is a prevalent morbidity with a typically early onset and chronic manifestation (Bruce et al., 2005). Symptoms revolve around an intense, persistent (anticipatory) fear of social and performance situations that is usually accompanied by increased autonomic arousal (McTeague et al., 2009) and results in subsequent avoidance behavior (Fehm et al., 2008). While avoidance is an undisputed maintaining mechanism in all anxiety disorders, it cannot fully explain the persisting nature of SAD (Hirsch and Clark, 2004). A large number of studies have pointed out that hypervigilance toward threatening information may represent a key mechanism contributing to the maintenance of this disorder (Staugaard, 2010). It has been suggested that social stimuli, especially faces signaling threat or disapproval, are particularly salient for individuals with social anxiety (Rapee and Heimberg, 1997), possibly as a result of the inherent biological preparedness (Öhman and Mineka, 2001b) and aversive learning experiences. Accordingly, socially anxious individuals have been shown to exhibit an attentional bias toward angry faces in visual attention paradigms (Horley et al., 2004; Pineles and Mineka, 2005; Klumpp and Amir, 2009) as well as enhanced neural reactivity toward angry expressions in limbic and extrastriate visual areas compared to healthy controls (Stein et al., 2002; Straube et al., 2005; Phan et al., 2006; Klumpp et al., 2012).

Enhanced unconscious threat processing is a possible mechanism underlying cognitive biases in anxiety disorders, as they may impact later processing stages and engender affective, cognitive as well as behavioral phenomenology, thus giving rise to the overgeneralization of fear (Dunsmoor et al., 2011). Although numerous studies have examined subliminal threat processing in other anxiety disorders (Brooks et al., 2012), only few studies have addressed this issue in socially anxious populations using disorder-specific stimuli, i.e., threatening faces. Empirical evidence, which mostly stems from analogous group studies, supports the notion that (social) anxiety is associated with altered early visual processing (Li et al., 2008), engagement and guidance of attentional resources (Mogg and Bradley, 2002; Holmes et al., 2009), enhanced subcortical response (Bishop et al., 2004; Phan et al., 2006; Vizueta et al., 2011) and may affect subsequent social judgments (Li et al., 2008) when the threatening stimuli are presented under conditions of restricted awareness.

Our research group has previously established a paradigmatic approach to investigate how subliminal threat cues may affect perceptual decisions (Jusyte and Schönenberg, 2013). In a series of experiments, healthy volunteers made affective judgments of morphed affective stimuli that were blends of neutral and angry expressions. Subliminal cues resulted in biased affective judgments of the morphed stimuli (i.e., more “angry” responses) only when the subliminal stimulus was angry and had been previously paired with an aversive experience. These results indicated that an acquisition of a perceptual bias to subliminal threat occurs only when the negative primes were paired with aversive outcomes in a previous conditioning procedure, which may mirror fear acquisition in real-world contexts. As highlighted earlier, patients with SAD represent a group with an especially pronounced bias to threatening facial expressions associated with alterations in preattentive processing. In contrast to healthy individuals, social anxiety may be associated with an increased salience of angry faces to such an extent, that even an unconscious “hint” of hostility may be enough to distort visual processing, resulting in a perceptual bias for anger even without prior conditioning, possibly due to prior aversive conditioning in real-world contexts. SAD patients may be perceptually more vulnerable to the biasing effects of unconscious threat cues, which could form the basis of affective, cognitive and behavioral symptoms in social anxiety.

The present study aimed to investigate this issue in individuals with SAD. Specifically, we were interested in whether subtle signals of threat that are presented under conditions of restricted awareness would result in biased performance on a subsequent affective judgment task. We expected SAD patients to make more “angry” responses if the preceding subliminal stimulus was angry as opposed to neutral, but healthy control participants were not expected to show this effect. The perceptual bias in the SAD group was expected to be larger for ambiguous mask stimuli (morphed facial expressions ranging between angry and neutral) due a larger susceptibility to biasing effects of the subliminal cues. In accordance with the affective judgment, we expected faster reaction times (RTs) for unambiguous as opposed to ambiguous mask stimuli for both groups and a facilitation of visual processing reflected in lower RT latencies for the subliminal threat condition in the SAD group only. These effects would provide further support for enhanced unconscious threat processing in SAD individuals and may have important implications for the development of new treatment strategies.

## MATERIALS AND METHODS

### PARTICIPANTS

Social anxiety disorder and control group participants were recruited via an electronic announcement, addressing all undergraduate students of the University of Tübingen who either experience anxiety in social interactions or have no interactional difficulties. Interested individuals were then invited for participation and completed a self-report battery of social anxiety measures and were administered a clinical interview in order to confirm the SAD/healthy control group status. All participants completed questionnaire diagnostics using German versions of several questionnaires assessing dimensional severity of social anxiety. *Social Interaction Anxiety Scale (SIAS)* was used to assess the anxiety experienced in social interactional situations; *Social Phobia Scale* (*SPS*; Mattick and Clarke, 1998; Stangier et al., 1999) was employed to measure levels of anxiety when individuals are scrutinized by others, and *Liebowitz Social Anxiety Scale* (*LSAS*; Liebowitz, 1987; Stangier et al., 2003) was used to assess the range of social interaction and performance situations that social phobics may fear/avoid. Furthermore, a structured interview [Mini International Neuropsychiatric Interview (MINI; Sheehan et al., 1998)] was administered by trained psychologists in order to validate the clinical diagnosis of SAD and to ensure the diagnosis-free status of healthy control participants. Exclusion criteria for the SAD participants were: a history of or current disorder of the schizophrenic or bipolar/manic spectrum, a diagnosis of borderline or antisocial personality disorder as well as awareness of the subliminal stimulus prime as assessed in the recognition task. In the healthy control group, exclusion criteria were a current psychopathology or a history thereof as well as awareness of the subliminal stimulus. Two participants from the SAD group and three controls were excluded due to their performance on the recognition task, which indicated that they were aware of the subliminal prime. The final sample of consisted of 23 SAD subjects and 23 healthy controls (see **Table 1** for more details). Subjects signed an informed written consent and received monetary compensation for participation. All experiments reported here were approved by the local ethics committee and are in accordance with the Declaration of Helsinki.

**Table 1:** Demographic and control measures.

	Controls *N* = 23		gSAD *N* = 23		Statistics
Age	23.48 (3.57)		23.96 (3.72)		*t*(44) = 0.445; n.s.
Female	55.5%		65.2%		*χ_(1)_^2^* = 0.365; n.s.
LSAS	14.56 (9.47)		75.30 (21.04)		*t*(44) = 11.17; *p*< 0.05
SIAS	10.33 (4.31)		43.30 (12.53)		*t*(44) = 18.75; *p*< 0.001
SPS	5.28 (8.25)		35.78 (12.95)		*t*(44) = 7.92; *p*< 0.05
*d*′	0.24 (0.64)		0.06 (0.60)		*t*(44) = -1.00; n.s.
CR correct	5.60 (2.16)		5.27 (1.86)		*t*(44) = -0.50; n.s.
CR error	5.47 (2.16)		5.11 (1.89)		*t*(44) = -0.58; n.s.


*The data represented in the table refers to means and SDs for each measure (in parentheses). LSAS = Liebowitz Social Anxiety Scale; SIAS = Social Interaction Anxiety Scale; SPS = Social Phobia Scale; CR = confidence rating.*

### MATERIALS

#### Facial stimuli

**FIGURE 1:**
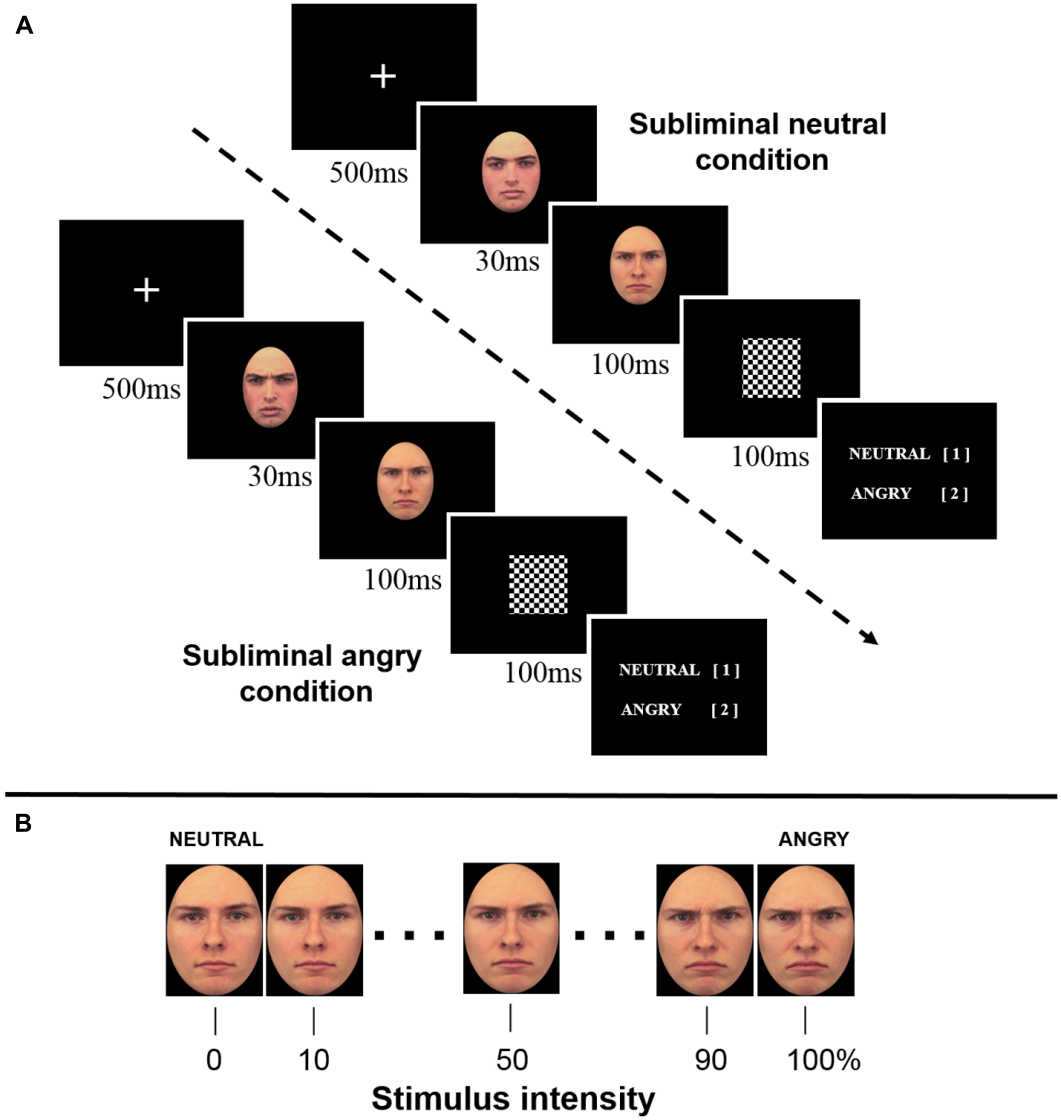
**(A)** Temporal trial structure for the perceptual decision task. **(B)** A stimulus set of one model identity parametrically varied in 10% increments ranging from neutral to angry.

Angry and neutral facial expressions of seven male models from the Karolinska Directed Emotional Faces database (Goeleven et al., 2008) were selected for the stimulus material. We only included models who depict anger without opening the mouth or baring teeth in order to limit the confounding effects of visual features in the masking procedure (Calvo and Nummenmaa, 2008). This resulted in a total of 14 color pictures (7 models × 2 expressions), which were edited in order to match the basic visual features (luminance, color) and size (cropping with an oval mask) using Adobe Photoshop CS4. This was necessary in order to achieve maximum masking efficiency. The emotional expression was parametrically varied using a morphing procedure (FantaMorph software, Abrosoft, Beijing, China) in which angry and neutral expressions of the same model were blended together. This resulted in a set of 11 intensity levels (10% increment steps) of angry expressions ranging from 0% (neutral) to 100% (angry) for each model (**Figure 1B**). One model identity was randomly selected for the subliminal stimulus set (unambiguous neutral and angry expressions). The stimulus material for the perceptual judgment task consisted of graded expressions of the remaining models (6 remaining models × 11 intensity levels), which were used as mask stimuli and two subliminal stimuli (neutral and angry expression of a randomly selected model identity). Visual stimuli were delivered via Presentation software (Version 14.5) throughout all phases of the experiment. Face stimuli (300 × 375 pixel) were presented in the center of an 19′′ CRT monitor against a black background.

### PROCEDURE

After providing written informed consent, participants completed the questionnaires and the diagnostic interview. The subsequent experimental procedure included three consecutive steps: In the first step, the participants were exposed to the subliminal stimulus set in order to establish a comparability to the original experimental design from our previous studies (Jusyte and Schönenberg, 2013). Next, the participants performed the perceptual decision task. In the third step, the participants’ ability to perceive the subliminal stimulus was assessed in order to ensure that all subjects were unaware of the subliminal stimulus condition.

#### Step I: exposure

During the exposure phase, neutral (50% of the trials) and angry expressions of one model identity (which later served as the subliminal stimulus pair) were presented a total of 20 times in pseudo-randomized order with no more than three identical trials in a row. The temporal structure for the exposure trials was as follows: an angry/neutral face was presented for 4 s, followed by 1 s inter-trial-interval (ISI, blank screen). Participants were instructed to pay close attention to the visual stimuli in order to “get acquainted with the stimulus material”^1^.

#### Perceptual decision task

The task for the participants was to indicate whether a briefly presented face stimulus was angry or neutral via a button press. The participants were not informed about the subliminal stimulus presentation and were instructed to react as quickly and accurately as possible. Trials were organized in blocks with either a subliminal presentation of angry or neutral stimulus on every trial throughout the whole block. One block consisted of 22 trials in which a subliminal stimulus was immediately masked by a supraliminal presentation of a mask stimulus. Per block, 11 intensity levels of two different models were presented once in random order. A total of six blocks were necessary in order to present all intensity levels of each model once with a preceding subliminal neutral as well as angry prime. Four repetitions or a total of 528 trials (6 models × 11 intensities × 2 subliminal stimuli × 4 repetitions) were presented during the experiment. Block and trial order was randomized for each repetition and participant. The temporal trial structure was as follows: The trial began with a fixation cross (500 ms, centered) followed by a subliminal angry or neutral stimulus (30 ms) and immediately replaced by a 100 ms presentation of the mask, which was then followed by a 100 ms checkered stimulus (**Figure 1A**) and finally the perceptual decision task. After the participants’ response, the next trial began after a 1 s ISI.

#### Recognition task

A major issue in all paradigms investigating subliminal processing is the difficulty ensure that these stimuli were not consciously perceived (Pessoa, 2005). In order to address this issue, several steps were undertaken (Li et al., 2008). First of all, the participants had no notion of the subliminal stimulus condition. During the experimental task, the subliminal stimuli were presented for merely 30 ms and backwardly masked by a stimulus with very similar perceptual properties. Furthermore, in a recognition task following the experiment, we assessed both subjective and objective awareness of the subliminal prime in a perceptual decision task and a subsequent confidence rating.

Before the recognition task, participants were debriefed about the subliminal stimulus presentations and were instructed to indicate whether the first, brief stimulus was neutral/angry and to ignore the mask. Following the perceptual decision, the participants were asked to indicate how confident they were that they answered correctly on a scale ranging from 1 (not sure at all) to 10 (completely confident). The confidence rating was chosen as a subjective measure of awareness. In a total of 36 randomized trials (6 models × 2 subliminal stimuli × 3 repetitions), intermediate intensity pictures (50%) of each of the six models from the experimental task were presented as face masks and preceded by either a subliminal angry or neutral stimulus. The (temporal) trial structure was identical to the perceptual decision task with the exception of the confidence rating. *d*′ scores were computed as objective indices of awareness. Both subjective and objective awareness of the subliminal stimulus condition were taken into account and only subjects who were considered unaware in both respects were included in the final analysis. Subjects were considered unaware and included in the analysis if they produced a *d*′ score between 1 and -1 (*d*′ range = +/-3.829; *d*′ = 0 indicates no discriminatory ability) and did not exhibit significantly higher confidence ratings on correct vs. erroneous responses in the recognition task.

## RESULTS

### SAMPLE

Demographic and psychopathological description of the final sample is displayed in **Table 1**. There were no significant differences with regard to age, gender, educational status, objective/subjective indices of awareness of the subliminal stimulus between groups. The SAD group scored significantly higher on all three dimensional measures of social anxiety (LSAS, SIAS, SPS) than the control group. None of the control group participants was diagnosed in the structured interview. All experimental group participants fulfilled the categorical diagnostic criteria for social phobia.

### RECOGNITION TASK

*d*′ scores were computed for each participant. Participants who outperformed the criterion range were excluded from the analysis (two control group and three SAD group participants). A one-sample *t*-test for the final sample revealed no significant difference from chance level for neither the control [*t*(22) = 1.84; *p* > 0.05] nor the SAD [*t*(22) = 0.49; *p* > 0.1] group; the analysis over collapsed data across groups also did not reach significance [*t*(45) = 1.67; *p* > 0.1]. To investigate the subjective awareness of subliminal stimulus condition, we computed paired-sample-*t*-tests regarding confidence ratings on the correct vs. incorrect responses in the recognition task for each group [SAD: *t*(22) = 1.10; controls: *t*(22) = 0.53; *p*s > 0.1], yielding no significant differences (see **Table 1** for more details). The results showed that the subjects had virtually no awareness for the subliminal stimulus condition.

### PERCEPTUAL DECISION TASK

The data analysis for the perceptual decision task was conducted in several steps: Firstly, we computed an analysis in order to investigate the potential perceptual bias. For this purpose, an analysis was computed for each group with total values reflecting the mean number of “angry” responses for each subliminal stimulus type and mask stimulus intensity. In a second step, we aimed to explore potential group differences in the perceptual bias related to subliminal stimulus type by employing *d*′ scores. This type of analysis is a more sophisticated way to examine the relative biases in perception for angry as opposed to neutral primes and has the advantage of reflecting the perceptual bias in a single value, thereby reducing the complexity of the model. Lastly, in order to control for potential speed-accuracy trade-offs that may be associated with the observed effects, we conducted an analysis of RT data.

#### Perceptual bias

**FIGURE 2:**
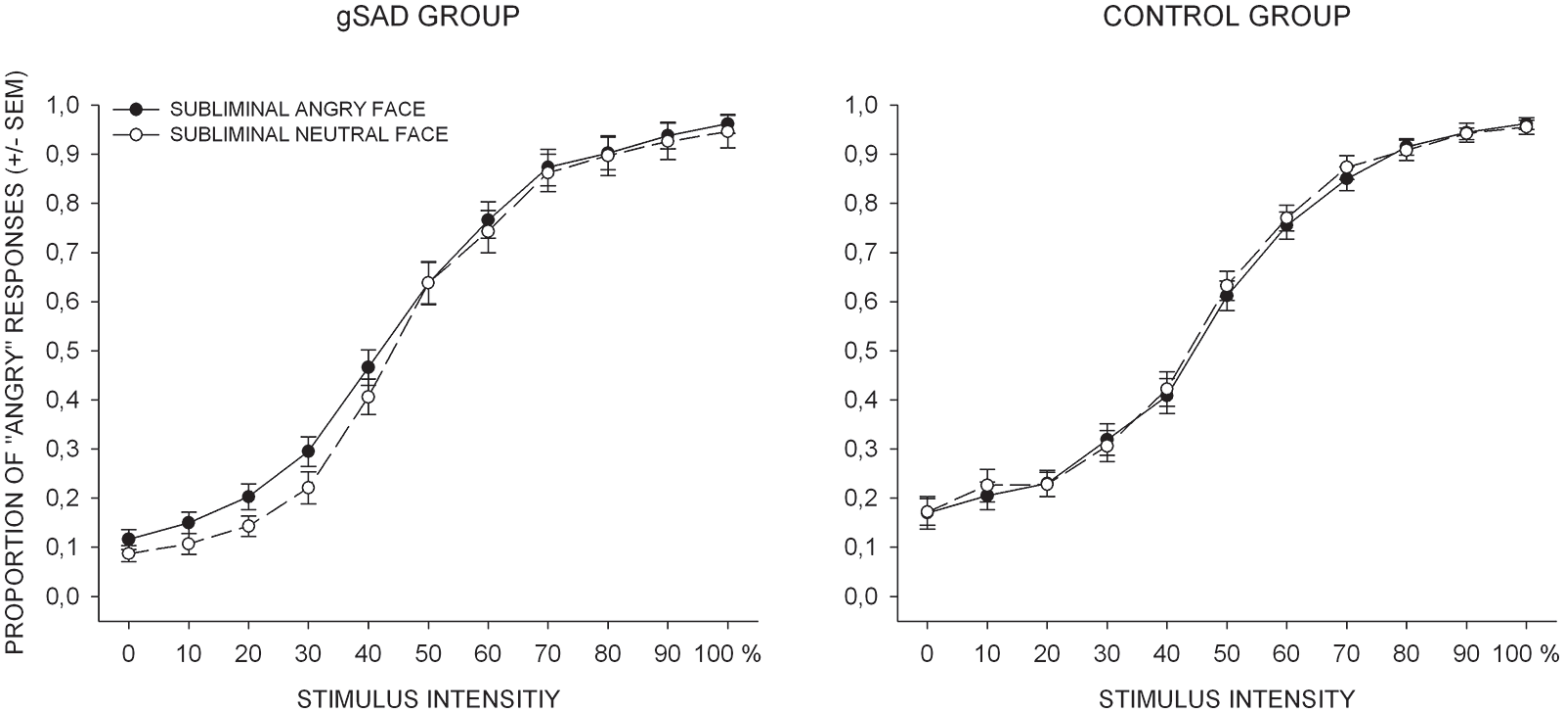
**Behavioral results for the perceptual judgment task.** The percentage of “angry” responses is plotted against stimulus intensity ranging from neutral (0) to angry (100). The dark circles and solid lines represent an angry subliminal stimulus, the white circles and dashed lines represent a neutral subliminal stimulus. gSAD, generalized social anxiety disorder; SEM standard error of mean.

In order to investigate the perceptual bias, an initial repeated-measures ANOVA with two within-subjects factors (subliminal stimulus type and intensity) as well as one between-subjects factor (group) was conducted using mean proportion of “angry” responses for condition and intensity level. The results indicated a main effect of stimulus intensity [*F*(10, 440) = 572.32; *p* < 0.001; $\eta_{p}^{2}$ = 0.93], which was further qualified by a significant condition × group [*F*(1, 44) = 572.32; *p* < 0.05; $\eta_{p}^{2}$ = 0.10] and a group × intensity interaction on a statistical trend level [*F*(10, 440) = 1.80; *p* < 0.10; $\eta_{p}^{2}$ = 0.04]. To further investigate the interaction effects, separate 2 (subliminal stimulus type) × 11 (intensity levels) repeated-measures ANOVA were computed (**Figure 2**) for each group. For the control group, there was a significant effect of stimulus intensity [*F*(10, 220) = 292.08; *p*< 0.001; $\eta_{p}^{2}$ = 0.93], but neither subliminal stimulus type [*F*(1, 22) = 0.23; *p* > 0.1; $\eta_{p}^{2}$ = 0.01] nor interaction [*F*(10, 220) = 0.44; *p* > 0.1; $\eta_{p}^{2}$ = 0.02] reached significance. The SAD group, however, showed a significant effect of both intensity [*F*(10, 220) = 282.67, *p* < 0.001, $\eta_{p}^{2}$ = 0.93] and subliminal stimulus type [*F*(1, 22) = 7.05; *p* < 0.05; $\eta_{p}^{2}$ = 0.24], as well as an interaction effect [*F*(10, 220) = 1.93; *p* < 0.05; $\eta_{p}^{2}$ = 0.08]. Paired-sample *t*-tests (subliminal angry vs. neutral stimulus) were computed in order to further qualify the interaction effect, yielding significant differences at the first five intensity levels (all *p*s < 0.05). Thus, the results indicate that only SAD group subjects tended to make more “angry” responses when the subliminal stimulus was angry.

In order to investigate whether the differences in perceptual biases are evident between groups, an additional joint analysis was computed. Indices of bias for angry and neutral subliminal conditions (*d*′ scores) were computed in the same manner as for the recognition task. This resulted in 11 scores (intensity of the mask stimulus) for each experimental condition. Positive scores represent a bias for angry response rating of the mask stimulus in the subliminal angry relative to the neutral condition, and vice versa, while a *d*′-value around 0 represents no systematic bias. *d*′ scores were analyzed using a repeated-measures ANOVA (**Figure 4**) with one within-subjects-factor (Intensity) and one between-subjects-factor (Group). Neither stimulus intensity [*F*(10,440) = 0.53; *p* > 0.1; $\eta_{p}^{2}$ = 0.01] nor the intensity × group interaction reached significance [*F*(10, 440) = 1.01, *p* > 0.1; $\eta_{p}^{2}$ = 0.02]. However, there was a significant group effect [*F*(1, 44) = 4.34, *p* < 0.05; $\eta_{p}^{2}$ = 0.09]. Subsequent one-sample *t*-tests computed with a total mean *d*′ score over all 11 intensity levels revealed no significant differences from chance level for the control group [*M*= -0.03; SD = 0.21; *t*(22) = -0.63; *p*> 0.1], whereas the effect was significant for the SAD participants [*M*= 0.10; SD = 0.20; *t*(22) = 2.36; *p*< 0.05]. These results indicate that a systematic tendency for angry responses as a function of subliminal stimulus condition was only evident in SAD participants as opposed to the control group.

Furthermore, a correlation analysis was conducted to further investigate the relationship between the extent of perceptual bias in the perceptual decision task (mean *d*′ scores reflecting the relative tendency to rate mask stimuli as “angry” when the preceding subliminal stimulus was angry) and the objective awareness measure (sensitivity *d*′ scores reflecting the ability to discriminate between the subliminal stimulus conditions) obtained in the recognition task. There were no significant correlations between these two measures neither on the group level (SAD: *r* = 0.15; controls: *r* = -0.10), nor in the collapsed data (*r* = -0.03, all *p*s > 0.1).

#### Reaction time (Abrantes-Pais et al., 2007)

**FIGURE 3:**
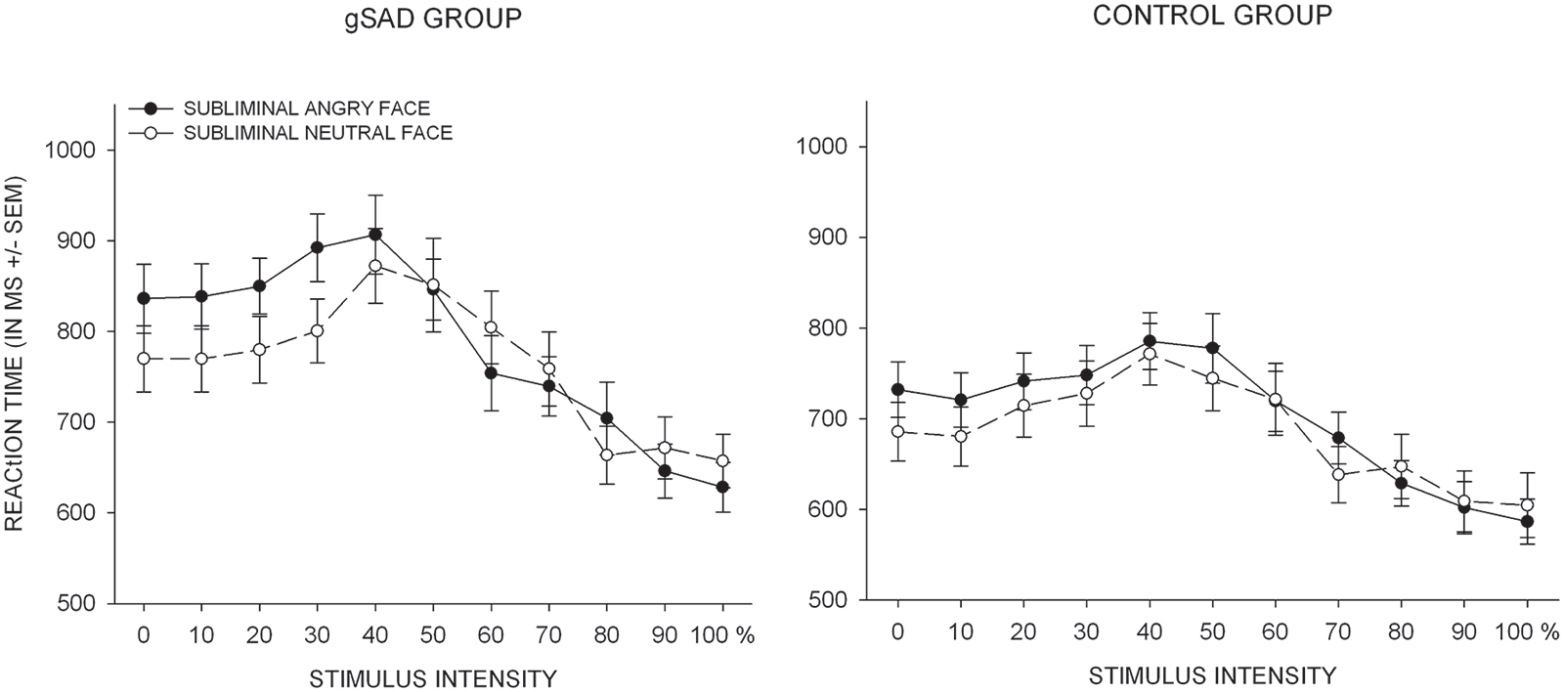
**Reaction time latencies for the perceptual judgment task.** Reaction times are plotted against stimulus intensity ranging from neutral (0) to angry (100). The dark circles and solid lines represent an angry subliminal stimulus, the white circles and dashed lines represent a neutral subliminal stimulus. gSAD, generalized social anxiety disorder. MS, milliseconds; SEM, standard error of mean

Reaction time latencies larger than three seconds were excluded from the analysis. The percentage of excluded trials was not significantly different between the control and SAD group [*M* = 6.39; SD = 10.48 and *M* = 3.13; SD = 4.24; *t*(44) = 1.36; *p* > 0.1]. An initial repeated measures ANOVA with the within-subjects factors subliminal stimulus type and intensity as well as one between-subjects factor (group) was conducted. The results yielded a main effect of stimulus intensity [*F*(10, 220) = 52.57; *p* < 0.001; $\eta_{p}^{2}$ = 0.54] as well as a significant effect of subliminal stimulus condition [*F*(10, 440) = 5.10; *p*< 0.05; $\eta_{p}^{2}$ = 0.10]. These main effects were further qualified by a condition × intensity interaction [*F*(10, 440) = 4.30; *p* > 0.01; $\eta_{p}^{2}$ = 0.09] and a condition × intensity × group interaction [*F*(10, 440) = 2.10; *p* > 0.05; $\eta_{p}^{2}$ = 0.05]. To further investigate the interaction effects, 2 (subliminal stimulus type) × 11 (stimulus intensity) repeated-measures ANOVAs were computed with mean RTs (**Figure 3**) for each group.

**FIGURE 4:**
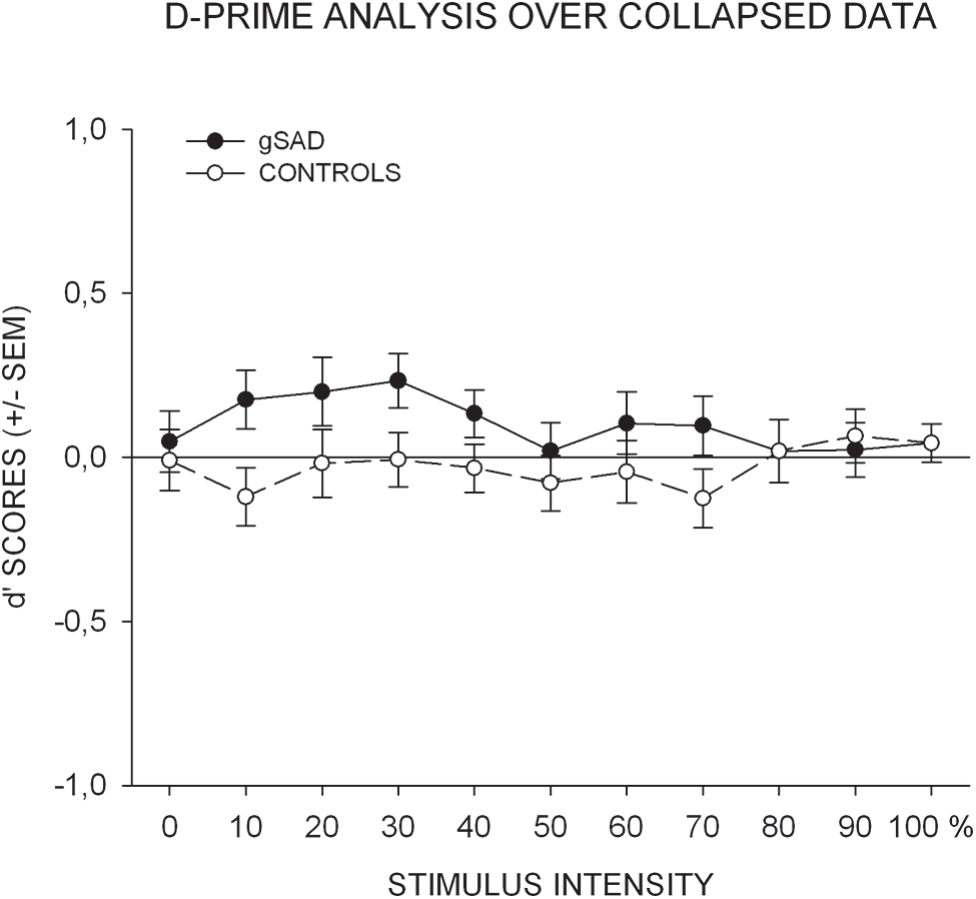
**The results of the *d*′ score analysis over collapsed data.** Average scores are plotted against stimulus intensity ranging from neutral (1) to angry (11). *d*′ scores > 1 indicate a bias for “angry” responses in the subliminal angry as opposed to neutral stimulus condition. gSAD, generalized social anxiety disorder; SEM, standard error of mean.

A significant effect of stimulus intensity emerged in the control group [*F*(10, 220) = 26.46; *p* < 0.001; $\eta_{p}^{2}$ = 0.55]; there was no significant effect of subliminal stimulus condition [*F*(1, 22) = 2.29; *p*> 0.1; $\eta_{p}^{2}$ = 0.09] nor did the interaction [*F*(10, 220) = 1.67; *p* > 0.05; $\eta_{p}^{2}$ = 0.07] reach significance. In the SAD group, there was a significant effect of stimulus intensity [*F*(10, 220) = 27.45; *p* < 0.001; $\eta_{p}^{2}$ = 0.55] and a significant interaction [*F*(10, 220) = 4.05; *p*< 0.001; $\eta_{p}^{2}$ = 0.16], but no significant effect of subliminal stimulus type [*F*(1, 22) = 2.8; *p*> 0.1; $\eta_{p}^{2}$ = 0.11]. Paired-sample *t*-tests (subliminal angry vs. neutral stimulus) were computed for the *post hoc* analysis in order to further investigate the interaction effect for each intensity level of the mask stimuli. The results revealed that SAD participants exhibited significantly higher RT latencies when the subliminal stimulus was angry vs. neutral at the first four intensity levels (all *ps* > 0.05). In both the SAD and the control group, a significant quadratic [*F*(1, 22) = 39.90; *p* < 0.001; $\eta_{p}^{2}$ = 0.64 vs. *F*(1, 22) = 79.24; *p* < 0.001; $\eta_{p}^{2}$ = 0.78) as well as linear trend [*F*(1, 22) = 47.17; *p* < 0.001; $\eta_{p}^{2}$ = 0.68 vs. *F*(1, 22) = 51.40; *p* < 0.001; $\eta_{p}^{2}$ = 0.70) emerged for the averaged RT data, which indicates an inverted U-shape pattern as well as lower RT latencies for unambiguous angry vs. neutral expressions.

## DISCUSSION

The present study investigated whether SAD patients are more susceptible to the biasing effects of threatening subliminal cues. The results of the perceptual judgment task showed that SAD subjects tended to make more “angry” responses regarding graded mask stimuli in trials with a preceding angry vs. neutral subliminal cue, while the proportion of “angry” responses did not vary as a function of subliminal stimulus condition in healthy subjects.

These results may reflect alterations in early visual processing, which possibly stem from hypersensitivity to threatening cues in associated subcortical structures (Straube et al., 2005; Phan et al., 2006; Stein et al., 2007). Accordingly, subliminal threat cues have been shown to elicit a robust neural response, particularly in anxiety-prone individuals (Li et al., 2008; Ball et al., 2012; Brooks et al., 2012). Several studies have investigated whether social phobia is associated with an increased sensitivity to facial expressions of threat by employing morphed stimuli of varying emotional intensity yielding conflicting findings (Richards et al., 2002; Mullins and Duke, 2004; Philippot and Douilliez, 2005; Joormann and Gotlib, 2006; Montagne et al., 2006; Rossignol et al., 2007; Schofield et al., 2007; Stevens et al., 2008; Garner et al., 2009; Heuer et al., 2010). Most of these studies failed to demonstrate that social anxiety is associated with a biased interpretation of emotion (Mullins and Duke, 2004; Philippot and Douilliez, 2005; Schofield et al., 2007) while one study reported a higher (Montagne et al., 2006) and another a lower (Joormann and Gotlib, 2006) threshold for the onset of negative emotion in facial expressions. The results of the present study are in line with previous literature which failed to find evidence for a biased interpretation of emotion in SAD, as our findings do not indicate a dramatically increased general perceptual sensitivity to angry expressions in SAD, but they do provide support for a vulnerability to the biasing effects of unaware stimuli. The hypersensitivity and earlier onset of hostile cue perception in facial expressions, of which the anxious individual may not even be aware, has the potential to cause anxious rumination and misinterpretation of the social partner’s facial expression, resulting in a cognitive overload and a failure to down-regulate these emerging misinterpretations by means of a top-down control.

Interestingly, the perceptual bias was observed at relatively low perceptual intensities of anger in mask stimuli (0–50% anger proportion) in the preceding subliminal angry vs. neutral stimulus condition. This finding is intriguing, because one would expect the biasing effect of subliminal cues to be most prominent at intermediate stimulus intensity levels of the mask stimulus due to their ambiguity. Our data shows that the SAD group is particularly sensitive to the biasing effects of the hostile subliminal stimulus even when the mask stimulus barely contains anger.

The overall results of RT latency revealed an inverted U-shape pattern with respect to stimulus intensity in both groups, reflecting lower RTs for unambiguous angry and neutral expressions and an increase at intermediate stimulus intensity levels. Hence, both groups exhibited peak RT latencies at intermediate intensity levels. This pattern may reflect judgment uncertainty associated with stimulus complexity, which can be assumed to be higher for ambiguous vs. prototypical expressions (Lim and Pessoa, 2008). Moreover, both groups exhibited faster RTs for unambiguous angry vs. neutral expressions. This pattern indicates a behavioral speeding effect for angry faces, which may reflect a prioritized processing of angry vs. neutral stimuli (Lim and Pessoa, 2008; Lee et al., 2009).

Furthermore, we hypothesized that the perceptual bias evident in the SAD group would also be associated with a behavioral speeding, i.e., faster RT latencies, in the subliminal angry vs. neutral condition. Our data did not provide support for this assumption; in fact, a contrary interactional effect emerged: SAD subjects tended to show higher RT latencies in the subliminal angry cue condition at low to intermediate intensity levels. Interestingly, the differential RT slowing corresponded closely with the intensity levels at which perceptual judgment bias for subliminal angry vs. neutral condition was most prominent. This may be due to the incompatibility between the prime and the masking stimuli that call for different response alternatives and result in a competition, which is considered to be a major determinant of prolonged RT and erroneous responses (Klapp and Hinkley, 2002; Praamstra and Seiss, 2005). Furthermore, the evidence regarding RT speeding for threatening faces in SAD patients appears to be rather inconsistent (Staugaard, 2010). While some studies report a behavioral facilitation for affective material (Becker, 2009; Lee et al., 2009; Olatunji et al., 2011), there is also a line of evidence demonstrating a behavioral interference, in particular for negative stimuli (Buodo et al., 2002; Pereira et al., 2006; Sommer et al., 2008; Pereira et al., 2010). Recent evidence has also uncovered the neural mechanisms underlying the interference effects of negative emotional stimuli on behavior (RT slowing), which may represent the basis of defensive behavioral responses such as freezing (Pereira et al., 2010; Pichon et al., 2012).

The present study extends our previous experimental work, which has some implications for the understanding of general mechanisms of affective stimulus processing as well as for the etiological models of anxious psychopathology. The affective judgment pattern observed in the SAD group strongly resembles the results obtained in Experiment 1 of our previous experimental series (Jusyte and Schönenberg, 2013). The behavioral data of healthy participants who performed the same judgment task after undergoing an aversive learning procedure, where the angry face (which later served as the subliminal stimulus in the affective judgment task) was paired with an aversive outcome, bears substantial similarity to the performance of SAD participants, who did not receive aversive conditioning. Therefore, the paradigm employed in our previous investigation with healthy participants may be an analog of the naturalistic process by which attentional vigilance in social anxiety develops, where an inherently negative stimulus is repeatedly paired with aversive experiences. To some extent, this may also reflect a natural and adaptive process by means of which individuals become more sensitive to facial displays of threat/dissaproval in those individuals with whom they associate unpleasant experiences. In future studies, it would be interesting to investigate whether other forms of experiential learning based on interactional outcomes, such paradigms involving social exclusion or inclusion experiences, would result in a similar sensitization toward subliminal threat stimuli.

These results of the present study also have some important implications regarding the development and maintenance of SAD. The data indicate that SAD patients exhibit an inherent anxious response pattern and appear to be sensitive to even very subtle signs of threat, which have the potential to guide volitional behavior. The fact that SAD participants do not require conditioning in order to unfold this sensitivity may be due to previous learning experiences in real life, in which facial expressions of anger or disapproval have acquired a potent signaling function. An angry face may represent such a highly potent signal of threat for social phobics that even a subtle “hint” of a hostile percept could suffice to bias early visual processing, resulting in a perceptual bias for “angry” responses even without prior conditioning, possibly due to prior aversive conditioning in real-world contexts.

Interestingly, both in this as well as our previous investigation using the same paradigmatic approach, we did not find evidence for a biased performance as a function of subliminal prime in healthy individuals, which contradicts a large number of studies from the priming literature (Murphy and Zajonc, 1993; Rotteveel et al., 2001; Winkielman et al., 2005; Dannlowski and Suslow, 2006; Almeida et al., 2013). On the other hand, not all studies have been able to replicate the threat processing advantage and conflicting evidence is reported over a variety of paradigms stemming from the attentional as well as perceptual unawareness literature (Pessoa, 2005; Pessoa et al., 2005, 2006; Bar-Haim et al., 2007; Purcell and Stewart, 2010; Lee et al., 2011). This may for one be due to the stimulus material employed in these studies. For instance, in an experimental series conducted by Calvo and Nummenmaa (2008), the authors concluded that not the emotional valence but certain salient physical features may underlie the processing advantage of emotional expressions in the face in the crowd paradigm. These salient features refer to the distribution of luminance in an emotional face caused by narrowing or widening the eyes, visibility of the teeth or opening the mouth. Paradigms for the investigation of subliminal threat processing may be even more vulnerable to these confounding effects. Considering the fact that many of the studies from the priming literature used characters rather than faces as masks, and the primes themselves were not cropped to remove areas such as hair in order to reduce contrast and target visibility, the reported priming effects may in part be due to a greater prime visibility. In addition, it is very hard to rule out this possibility due to the fact that most studies did not employ a valid awareness manipulation check to rule out this possibility. Some authors go so far as to say that priming effects may actually just reflect visual confounds caused by insufficient masking ability (Pessoa, 2005; Pessoa et al., 2005, 2006). However, a number of recent findings call these conclusions into question. For instance, studies that had employed extremely brief presentation times (17–20 ms) still found a reliable amygdalar signal to briefly presented threatening stimuli (Pegna et al., 2004; Whalen et al., 2004; Liddell et al., 2005; Williams et al., 2005; Ohrmann et al., 2007; Pegna et al., 2008). Hence, the presence or absence of behavioral priming effects may critically depend on the extent of such activation, which is likely why this study did find priming effects in individuals who have been shown to be particularly sensitive to displays of threat, namely SAD.

The present study has several strengths and limitations worth mentioning. Among the strengths are the homogenous SAD and the well-matched control group as well as the within-subjects repeated-measures design, which provides for a high statistical power of the obtained results. Furthermore, we employed highly homogenous stimulus material regarding color, luminance and the distribution of light and dark areas in the emotional faces, which allowed for a very efficient masking procedure. The assessment of subjective as well as objective awareness of the subliminal stimulus is recommended for investigations which employ subliminal primes (Pessoa, 2005) and has been followed in the present study. One limitation concerns our stimulus material, which included only one emotional expression, namely varying intensities of anger. Several studies have shown that socially anxious individuals exhibit alterations in the processing of facial expressions exhibiting not only overt aggression (anger) but also milder forms of hostile expression that signal disapproval, such as disgust and contempt (Stein et al., 2002; Amir et al., 2005; Phan et al., 2006). Hence, future studies should attempt to investigate how the present findings extend to other forms of hostile facial expression. Furthermore, although we made attempts to match the priming and masking stimuli on low-level visual features by excluding models with visible displays of teeth, the influence of such features cannot be entirely ruled out. For instance, two recent studies that used subliminal presentation using continuous flash suppression indicated that low-level features, such as spatial frequencies, may underlie emotion processing advantages observed in similar paradigms (Stein and Sterzer, 2012; Stein et al., 2014). Perhaps the strongest argument against this is that we found group differences between SAD and healthy controls; thus, the perceptual sensitivity to subliminal displays of anger was associated with a factor related to an inherently individual characteristic of one group. Although the role of low-level features cannot be entirely ruled out, we believe that it does not sufficiently explain all of the results obtained in this study. For future research working with backward-masking paradigms, we recommend to include a more rigorous and sophisticated control of low-level visual features such as adjustments and matching of root mean square contrast.

In addition, while enhanced visual processing due to direct projections from hyperactive subcortical structures is a likely mechanism that accounts for the present results, we did not test these assumptions using brain imaging techniques. Moreover, we cannot rule out that the observed effects are related to differences in response priming rather than shifts in perceptual sensitivity, that is, the prime could simply affect response criteria, rather than actual expression perception. Future studies that employ both imaging techniques and sophisticated experimental designs which allow to distinguish between response and perceptual biases are needed to understand the underlying mechanism. Finally, this study did not elucidate whether the enhanced perceptual sensitivity is part of a SAD symptom correlate or rather a marker for vulnerability. This issue should be elucidated in future research.

In summary, the present work provides further evidence for enhanced perceptual processing of threatening facial expressions in SAD individuals. These findings beg the question whether the bias observed in our study is stable and whether it can be modified by means of classical cognitive–behavioral intervention methods or new computer-based training approaches that target attentional processes (Bar-Haim, 2010). It is possible that modification of later processing stages, may have a synergic effect on the automatic processing stages, but the anxious perceptual processing style may also be stable, which would mean that anxious individuals would always remain prone to relapse into an anxious psychopathology. Incorporation of these aspects in psychoeducation and strengthening the patient’s ability to employ top-down strategies in order to counter the hyperactive threat detection system may be a useful strategy to down-regulate the hypersensitive perceptual threat processing. The present paradigmatic approach may be useful in future studies in order to elucidate these issues and could also prove to be a suitable outcome measure that reflects early information processing.

## Conflict of Interest Statement

The authors declare that the research was conducted in the absence of any commercial or financial relationships that could be construed as a potential conflict of interest.

## Footnotes

1 The present study is an extension of our previous experimental work. In that study, we also included a conditioning procedure and investigated how learning experiences may influence preattentive processing using the same paradigm (Jusyte and Schönenberg, 2013). In this prior investigation, we included a conditioning procedure during which the subliminal stimuli used in the experiment served as CS^+/-^. In order to compare the results to a condition in which no conditioning was applied, we included an exposure phase prior to the experiment. Hence, the exposure procedure in this study was included as means of ensuring comparability to our previous work with healthy participants.
